# Supplementary material for: School-based high-intensity interval training programs in children and adolescents: A systematic review and meta-analysis
Source: PLoS One. 2022 May 4;17(5):e0266427. doi: 10.1371/journal.pone.0266427 (PMC9067698; doi:10.1371/journal.pone.0266427)
Supplement: S1 File — (DOCX) [file pone.0266427.s001.docx]

**Supplement 1. Search Terms**

**THESAURAUS TERMS**

**MEDLINE (OVID) MeSH:**

Adolescent

Child

Pediatrics

High Intensity Interval Training

**EMBASE (OVID) EMTREE:**

Adolescent

Child

Pediatrics

High Intensity Interval Training

**CINAHL (EBSCOHOST) SH:**

Adolescence

Minors

Child

High Intensity Interval Training

**SPORTDISCUS (EBSCOHOST) SU:**

Adolescent

Child

Pediatrics

High Intensity Interval Training

**KEYWORDS**

**Part I – high intensity interval training**

| High | Intensity | Interval | Training | Or |
| --- | --- | --- | --- | --- |
| “ | “ | “ | Exercise | Or |
| “ | “ | “ | Activity | Or |
| “ | “ | “ | Activities | Or |
|  |  |  |  |  |
| High | Intensity | Training |  | Or |
| “ | “ | Exercise |  | Or |
| “ | “ | Activity |  | Or |
| “ | “ | Activities |  | Or |
| “ | “ | Physical | Activity | Or |
| “ | “ | “ | Activities | Or |
| “ | “ | Aerobic | Training | Or |
|  |  |  |  |  |
| High | Intensity | Intermittent |  | Or |
|  |  |  |  |  |
| Intense | Intermittent | Training |  | Or |
| “ | “ | Exercise |  | Or |
| “ | “ | Activity |  | Or |
| “ | “ | Activities |  | Or |
|  |  |  |  |  |
| Intense | Interval | Training |  | Or |
| “ | “ | Exercise |  | Or |
| “ | “ | Activity |  | Or |
| “ | “ | Activities |  | Or |
|  |  |  |  |  |
| HIIT |  |  |  | Or |
| HIIE |  |  |  | Or |
|  |  |  |  |  |
| Sprint | Interval | Training |  |  |

**Part II – children and adolescents**

Child Or

Children Or

Adolescent* Or

Adolescence Or

Pediatric* Or

Paediatric* Or

Teen* Or

Teenager* Or

Youth* Or

“Young People” Or

Juvenile* Or

Boy* Or

Girl*

**Part I AND Part II = Final Search**
