# Supplementary material for: School-based high-intensity interval training programs in children and adolescents: A systematic review and meta-analysis
Source: PLoS One. 2022 May 4;17(5):e0266427. doi: 10.1371/journal.pone.0266427 (PMC9067698; doi:10.1371/journal.pone.0266427)
Supplement: S2 File — (DOCX) [file pone.0266427.s002.docx]

**Supplement 3. Certainty of Evidence based on Grading of Recommendations, Assessment, Development and Evaluation (GRADE).**

|  | **Outcome** | **Risk of Bias** | **Inconsistency** | **Indirectness** | **Imprecision** | **Publication Bias** | **Large Effect Size** | **Overall** |
| --- | --- | --- | --- | --- | --- | --- | --- | --- |
| Body Composition | Waist Circumference | -1 | 0 | 0 | 0 | 0 | 0 | 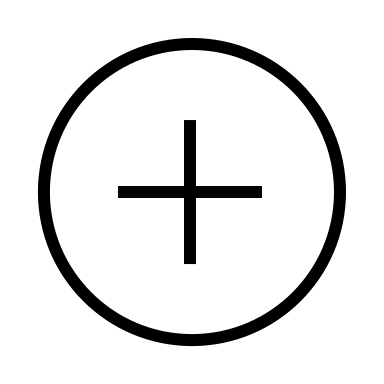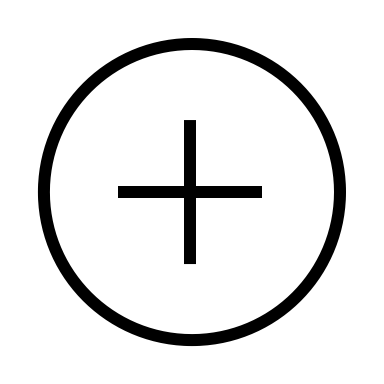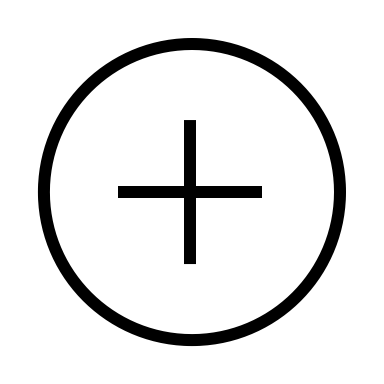 |
|  | Body Fat Percentage | -1 | -1 | 0 | 0 | -1 | +1 | 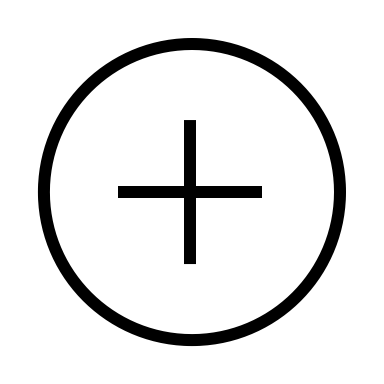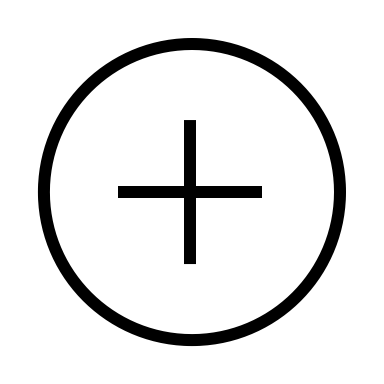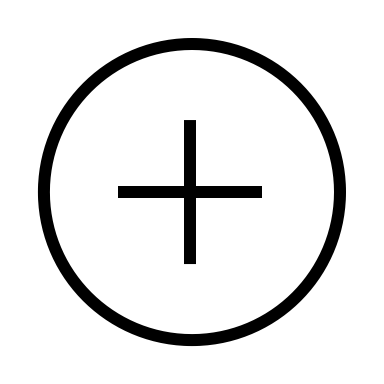 |
|  | Body Mass Index | -1 | -1 | 0 | 0 | -1 | +1 | 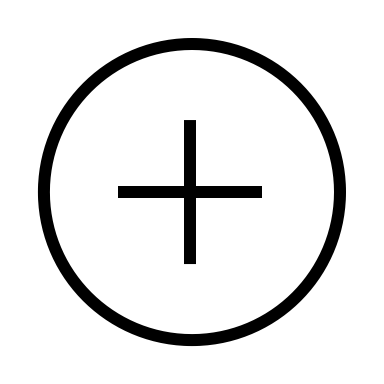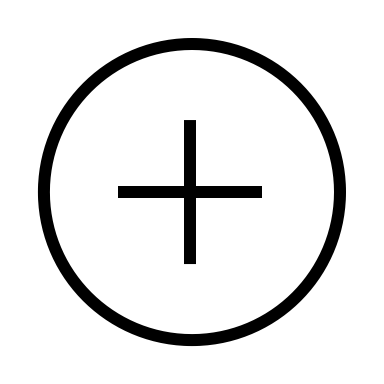 |
|  | Lean Mass | -1 | -1 | 0 | -1 | 0 | 0 | 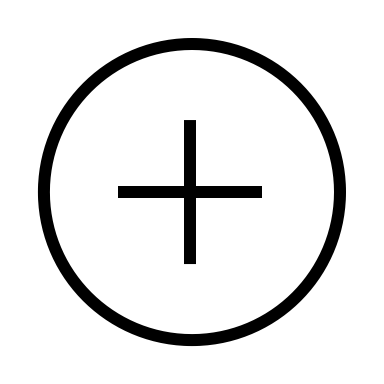 |
|  | Muscle Mass | -1 | 0 | 0 | -1 | 0 | 0 | 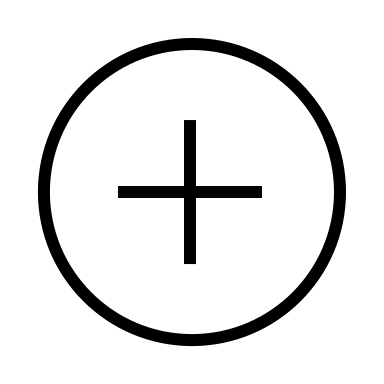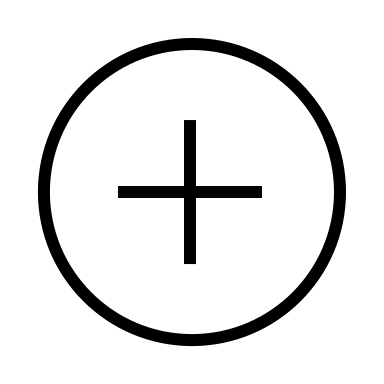 |
| Cardiovascular | Systolic Blood Pressure | -1 | 0 | 0 | 0 | 0 | 0 | 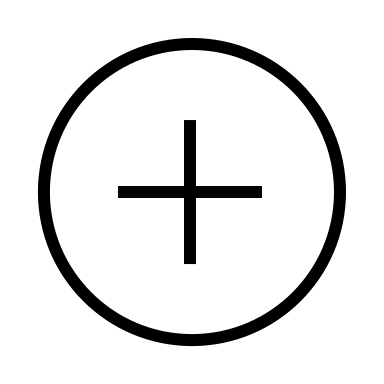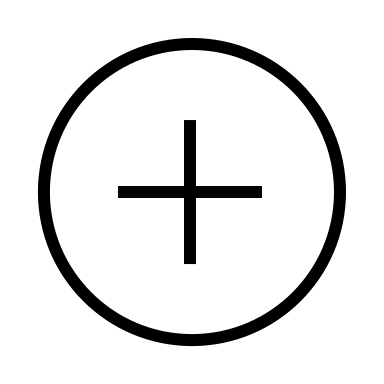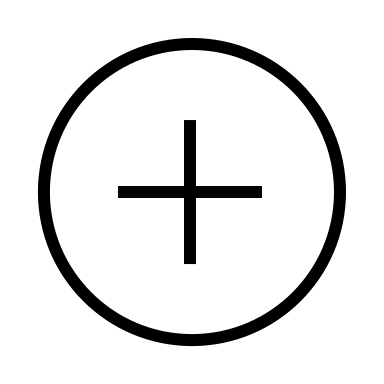 |
|  | Diastolic Blood Pressure | -1 | -1 | 0 | 0 | 0 | 0 | 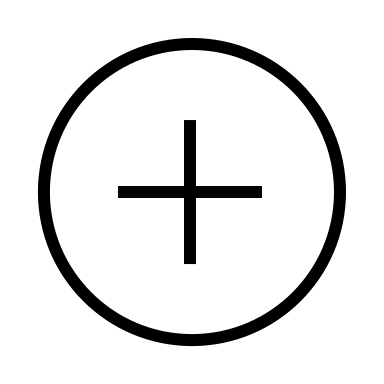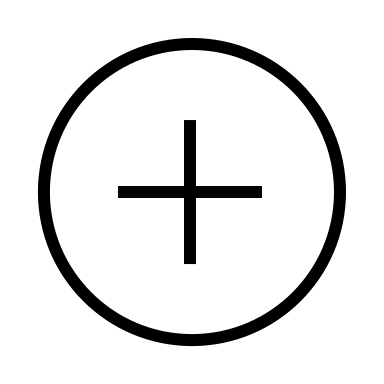 |
|  | Resting Heart Rate | -1 | -1 | 0 | 0 | 0 | 0 | 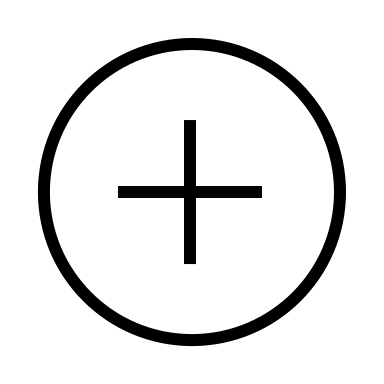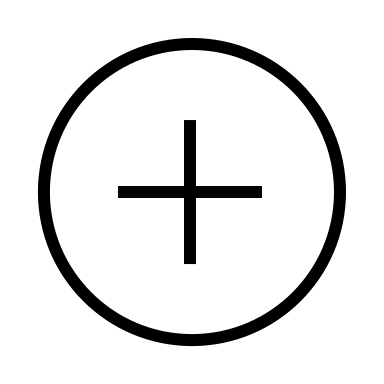 |
| Blood Profile | Glucose | -1 | 0 | 0 | 0 | 0 | 0 | 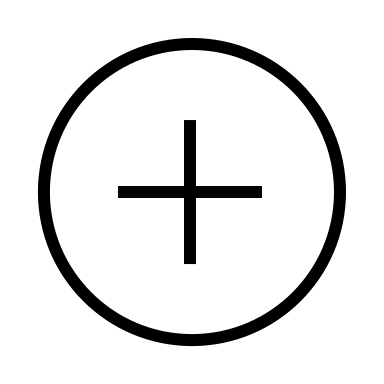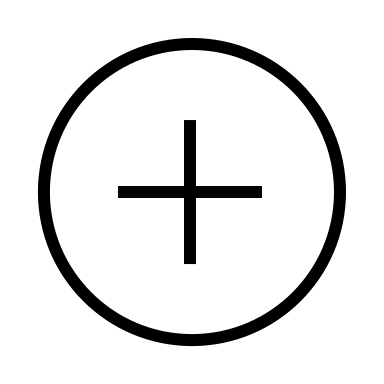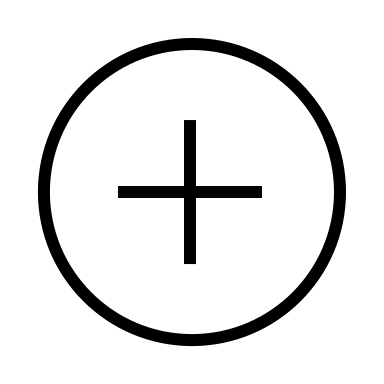 |
|  | Insulin | -1 | -1 | 0 | -1 | 0 | 0 | 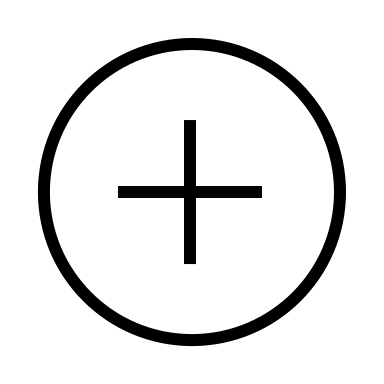 |
|  | HOMA-IR | -1 | 0 | 0 | 0 | 0 | 0 | 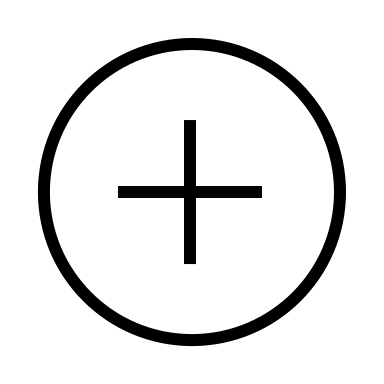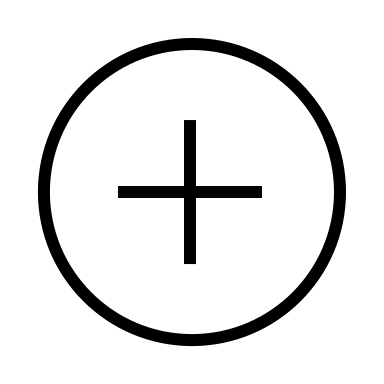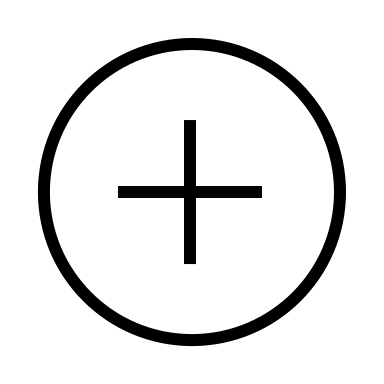 |
|  | Triglycerides | -1 | -1 | 0 | -1 | 0 | 0 | 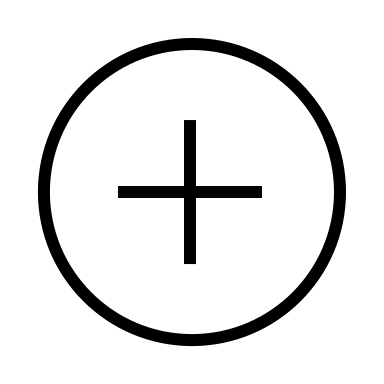 |
|  | Total Cholesterol | -1 | -1 | 0 | -1 | 0 | 0 | 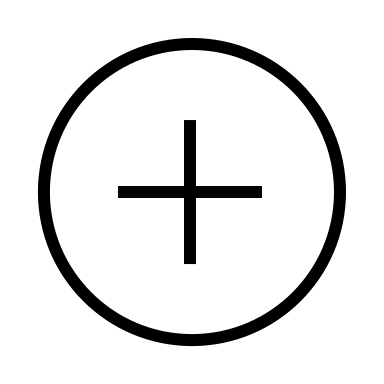 |
|  | High-density Lipoprotein | -1 | 0 | 0 | -1 | 0 | 0 | 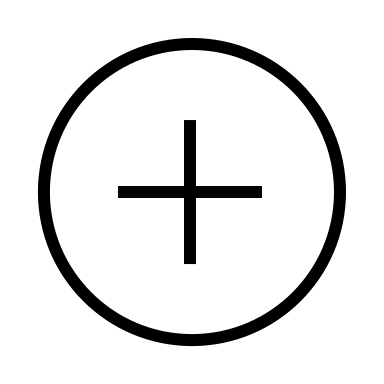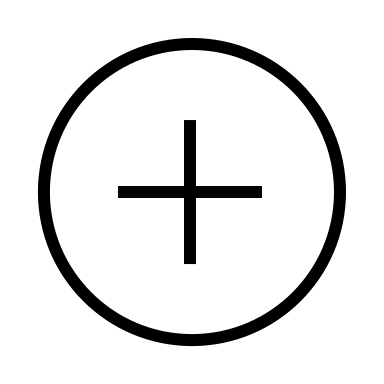 |
|  | Low-density Lipoprotein | -1 | 0 | 0 | -1 | 0 | +1 | 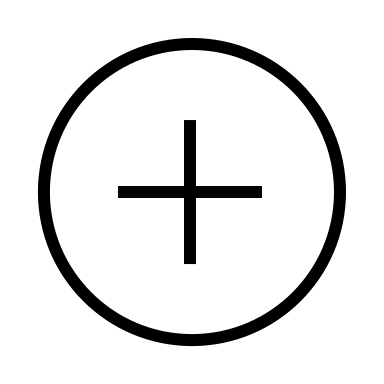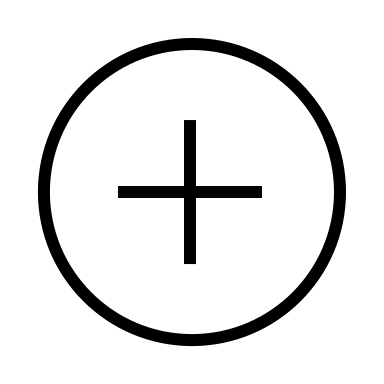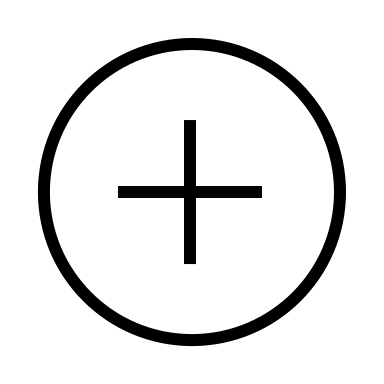 |
| Fitness | Cardiorespiratory Fitness | -1 | -1 | 0 | 0 | -1 | +1 | 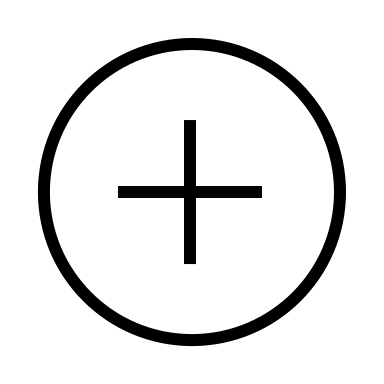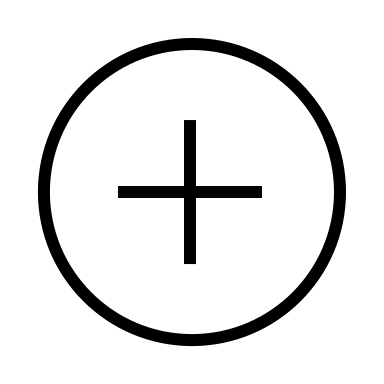 |
|  | Cardiorespiratory Fitness (VO2) | -1 | -1 | 0 | 0 | 0 | +1 | 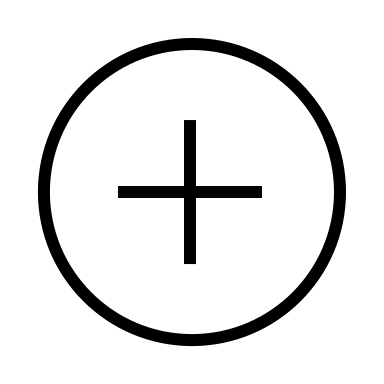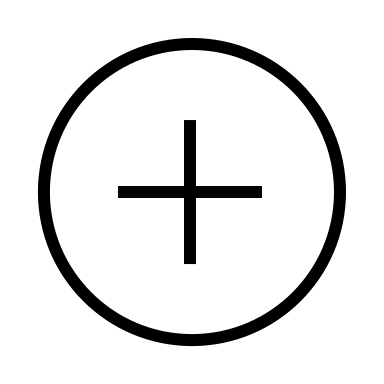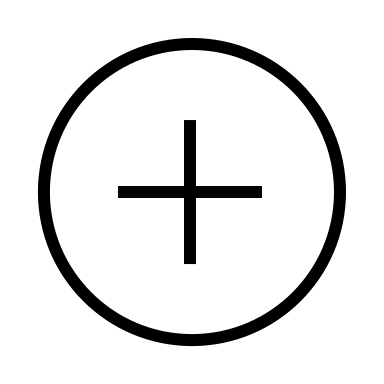 |
|  | Cardiorespiratory Fitness (shuttles) | -1 | -1 | 0 | -1 | -1 | +1 | 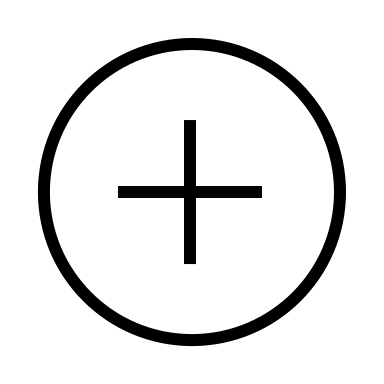 |
|  | Standing Long Jump | -1 | -1 | 0 | 0 | 0 | 0 | 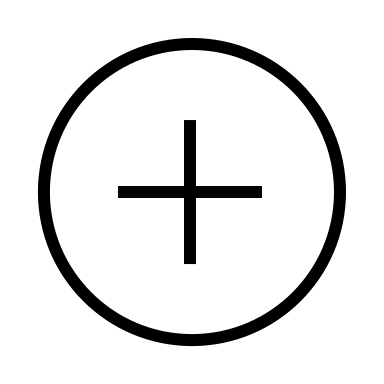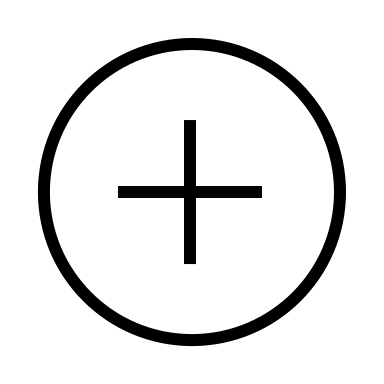 |
|  | Countermovement Jump | -1 | -1 | 0 | 0 | 0 | 0 | 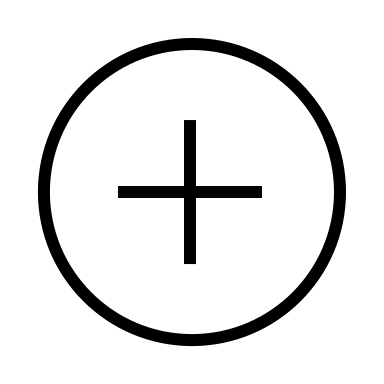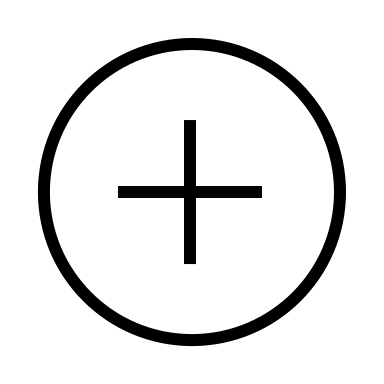 |

Certainty of Evidence classified as either “very low”, “low”, “moderate”, or “high”. The certainty could be downgraded due to a high risk of bias, inconsistency (unexplained heterogeneity), indirectness (lack of generalisability or external validity), imprecision (small sample size or wide confidence intervals), or the presence of publication bias. The certainty of evidence could be upgraded due to a large effect size.

HOMA-IR = homeostatic model assessment – insulin resistance.


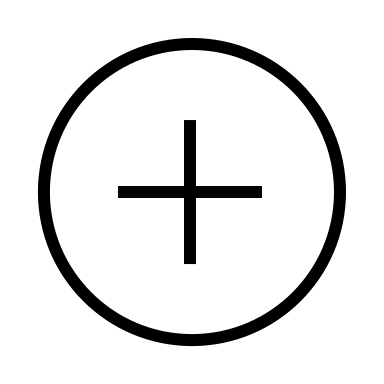
 = very low,
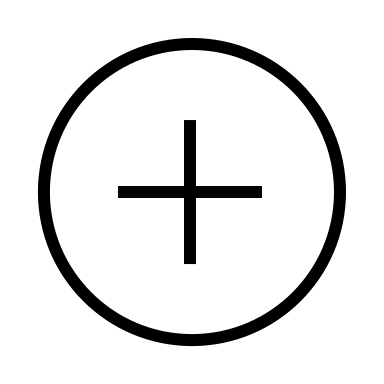

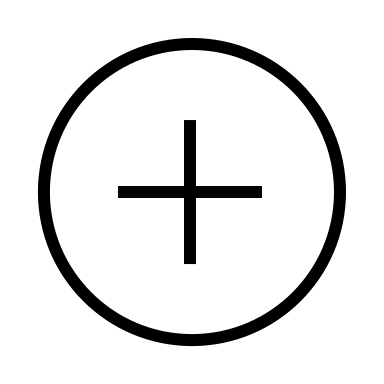
 = low,
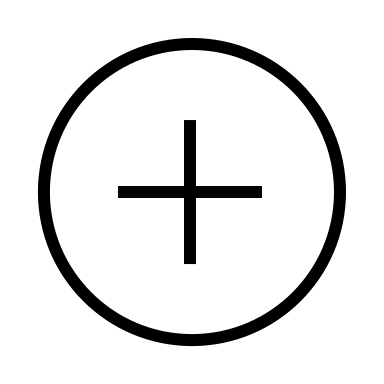

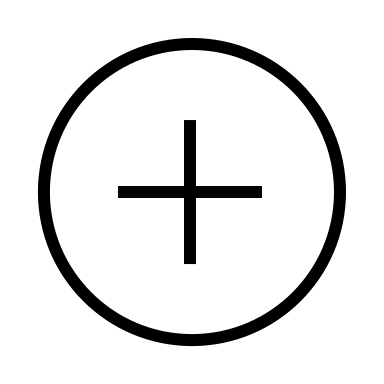

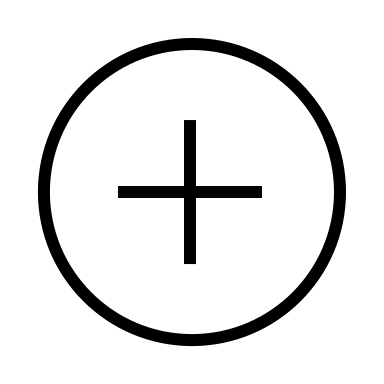
 = moderate,
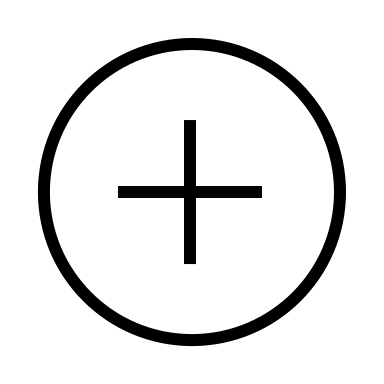

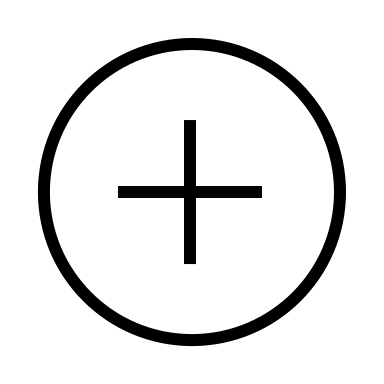

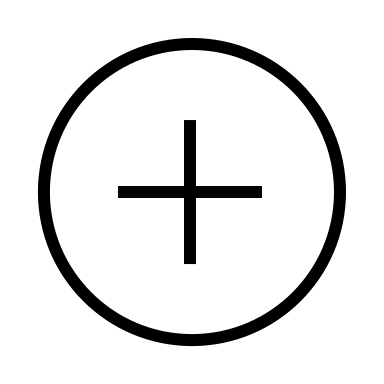

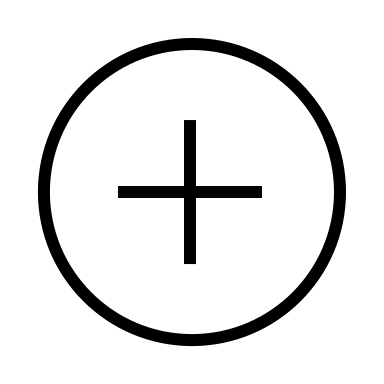
 = high
