# Supplementary material for: School-based high-intensity interval training programs in children and adolescents: A systematic review and meta-analysis
Source: PLoS One. 2022 May 4;17(5):e0266427. doi: 10.1371/journal.pone.0266427 (PMC9067698; doi:10.1371/journal.pone.0266427)
Supplement: S3 File — (PDF) [file pone.0266427.s003.pdf]

Supplement 3: Body Composition Forest Plots

1. Forest plot of high-intensity interval training versus control for waist circumference

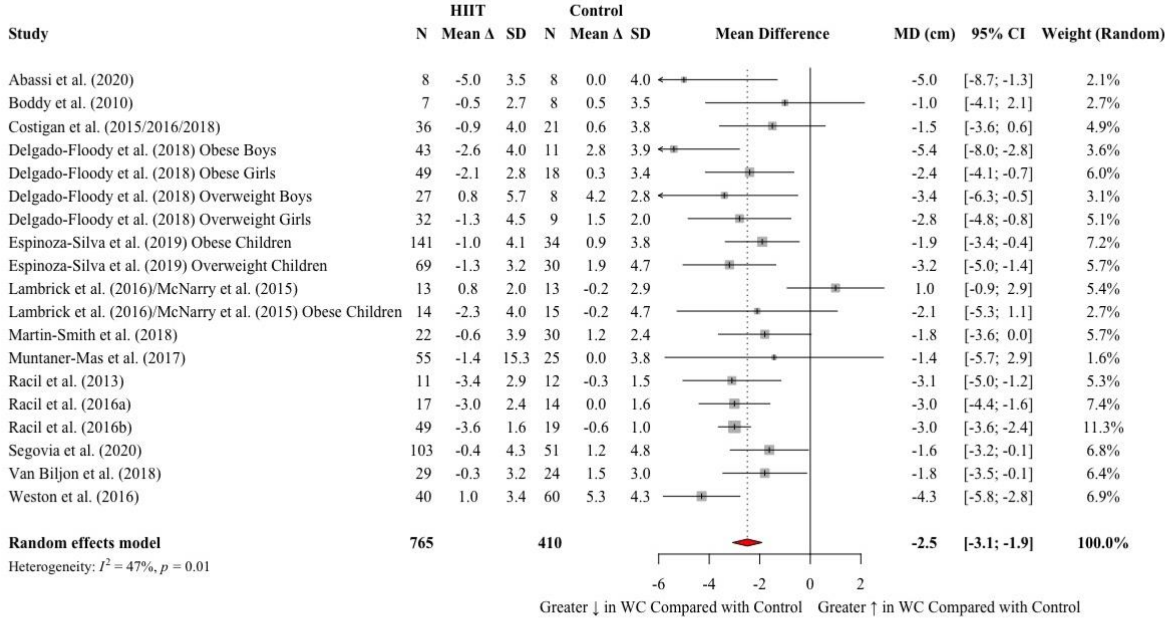

The mean difference between the change scores for high-intensity interval training (HIIT) and the control groups in centimetres

N = number of participants

Mean Δ = change score between pre- and post-tests

SD = standard deviation of the change score

MD = mean difference between the HIIT and control groups

CI = confidence interval

WC = waist circumference

I^2 = variation across studies due to heterogeneity rather than chance

2. Forest plot of high-intensity interval training versus control for body fat percentage

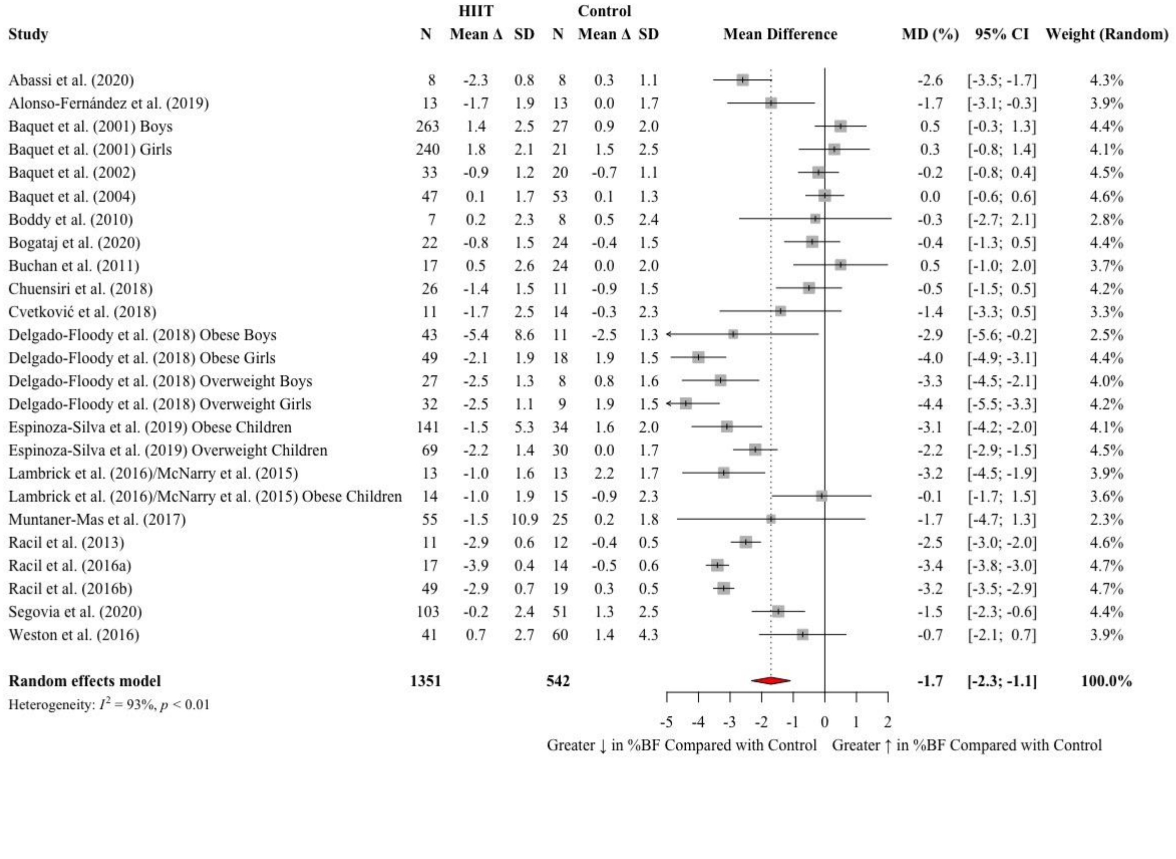

The mean difference between the change scores for high-intensity interval training (HIIT) and the control groups in percentage
N = number of participants
Mean Δ = change score between pre- and post-tests
SD = standard deviation of the change score
MD = mean difference between the HIIT and control groups
CI = confidence interval
%BF = percentage of body fat
I^2 = variation across studies due to heterogeneity rather than chance

3. Forest plot of high-intensity interval training versus control for body mass index

Study	HIIT N	HIIT Mean Δ	HIIT SD	Control N	Control Mean Δ	Control SD	SMD	95% CI	Weight (Random)
Abassi et al. (2020)	8	-2.1	1.1	8	0.1	1.3	-1.8	[-3.0; -0.5]	2.9%
Alonso-Fernández et al. (2019)	13	-0.2	0.5	13	-0.0	0.9	-0.2	[-1.0; 0.6]	3.5%
Baquet et al. (2001) Boys	263	0.3	0.8	27	0.7	0.8	-0.5	[-0.9; -0.1]	4.0%
Baquet et al. (2001) Girls	240	0.4	0.9	21	0.4	1.1	0.0	[-0.4; 0.4]	3.9%
Baquet et al. (2010)	22	0.1	0.4	19	-0.3	0.7	0.7	[0.1; 1.3]	3.7%
Boddy et al. (2010)	7	-0.5	0.6	8	0.2	0.8	-0.9	[-2.0; 0.2]	3.1%
Bogataj et al. (2020)	22	-1.0	0.4	24	-0.6	0.6	-0.8	[-1.4; -0.2]	3.7%
Buchan et al. (2011)	17	-0.3	0.5	24	-0.4	0.6	0.1	[-0.5; 0.8]	3.7%
Chuensiri et al. (2018)	26	-0.2	0.9	11	-1.0	0.9	0.9	[0.2; 1.6]	3.6%
Costigan et al. (2015/2016/2018)	40	-0.3	0.7	21	0.3	3.2	-0.3	[-0.8; 0.2]	3.8%
Cvetković et al. (2018)	11	-0.3	0.8	14	0.9	1.2	-1.1	[-1.9; -0.2]	3.4%
Delgado-Floody et al. (2018) Obese Boys	43	-1.0	1.0	11	0.2	1.2	-1.2	[-1.9; -0.5]	3.6%
Delgado-Floody et al. (2018) Obese Girls	49	-1.3	0.7	18	0.6	0.9	-2.3	[-3.0; -1.6]	3.6%
Delgado-Floody et al. (2018) Overweight Boys	27	-1.1	0.5	8	0.1	0.5	-2.4	[-3.4; -1.4]	3.2%
Delgado-Floody et al. (2018) Overweight Girls	32	-1.0	0.5	9	-0.3	0.9	-1.1	[-1.9; -0.3]	3.5%
Espinoza-Silva et al. (2019) Obese Children	141	-0.9	0.9	34	0.4	1.0	-1.4	[-1.8; -1.0]	3.9%
Espinoza-Silva et al. (2019) Overweight Children	69	-1.9	0.5	30	-0.0	0.9	-2.9	[-3.5; -2.3]	3.7%
Lambrick et al. (2016)/McNarry et al. (2015)	13	0.0	0.5	13	1.1	2.0	-0.7	[-1.5; 0.1]	3.5%
Lambrick et al. (2016)/McNarry et al. (2015) Obese Children	14	-0.1	0.9	15	-0.3	0.9	0.2	[-0.5; 0.9]	3.6%
Lubans et al. (2020)	329	0.1	0.5	310	0.1	0.5	0.0	[-0.1; 0.2]	4.1%
Martin et al. (2015)	18	-0.6	0.6	19	0.2	0.8	-1.1	[-1.8; -0.4]	3.6%
McNarry et al. (2020)	17	0.0	1.0	16	1.0	0.7	-1.1	[-1.9; -0.4]	3.6%
Muntaner-Mas et al. (2017)	55	0.2	0.6	25	0.4	0.5	-0.5	[-1.0; 0.0]	3.9%
Racil et al. (2013)	11	-0.4	0.1	12	0.0	0.0	-4.4	[-6.0; -2.8]	2.3%
Racil et al. (2016a)	17	-0.3	0.1	14	0.0	0.1	-2.1	[-3.0; -1.2]	3.3%
Racil et al. (2016b)	49	-0.4	0.1	19	-0.1	0.1	-4.1	[-4.9; -3.2]	3.4%
Van Biljon et al. (2018)	29	0.1	0.3	24	0.1	0.2	-0.0	[-0.6; 0.5]	3.8%
Weston et al. (2016)	41	0.3	0.6	60	0.1	1.2	0.2	[-0.2; 0.6]	4.0%
**Random effects model**	**1623**			**827**			**-0.9**	**[-1.3; -0.6]**	**100.0%**

Heterogeneity: $I^2 = 92\%$, $p < 0.01$

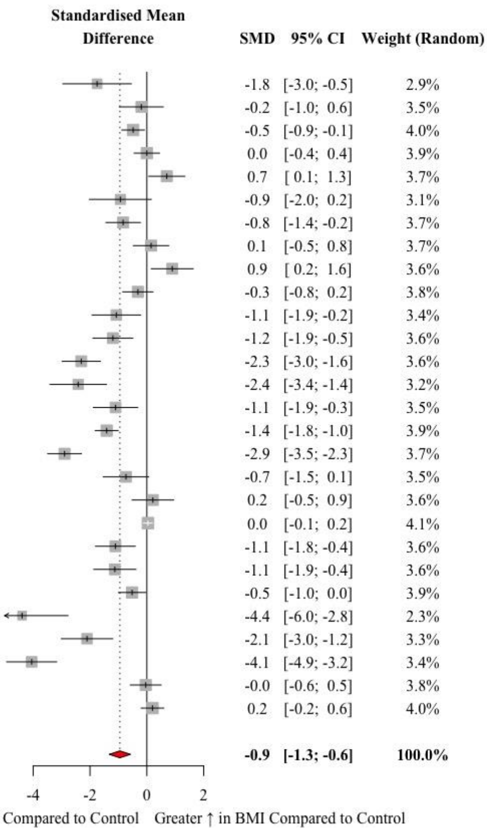

The standardised mean difference (SMD) between the change scores for high-intensity interval training (HIIT) and the control groups. SMD was used to account for numbers expressed as kg/m^2 and as z-scores.
N = number of participants
Mean Δ = change score between pre- and post-tests
SD = standard deviation of the change score
SMD = standardised mean difference between the HIIT and control groups
CI = confidence interval
BMI = body mass index
I^2 = variation across studies due to heterogeneity rather than chance

4. Forest plot of high-intensity interval training versus control for muscle mass

Study	HIIT N	HIIT Mean Δ	HIIT SD	Control N	Control Mean Δ	Control SD	MD (kg)	95% CI	Weight (Random)
Bogataj et al. (2020)	22	0.2	1.0	24	0.0	1.1	0.2	[-0.4; 0.8]	22.3%
Chuensiri et al. (2018)	26	1.4	2.0	11	1.2	2.0	0.2	[-1.2; 1.6]	7.3%
Cvetković et al. (2018)	11	0.7	1.5	14	1.8	1.6	-1.1	[-2.3; 0.2]	9.0%
Lambrick et al. (2016)/McNarry et al. (2015)	13	0.3	0.9	13	0.3	0.7	-0.0	[-0.6; 0.6]	21.7%
Lambrick et al. (2016)/McNarry et al. (2015) Obese Children	14	1.0	1.4	15	0.1	0.9	0.9	[0.0; 1.8]	14.7%
Weston et al. (2016)	41	0.5	1.2	60	-0.1	1.5	0.6	[0.1; 1.1]	25.0%
**Random effects model**	**127**			**137**			**0.3**	**[-0.2; 0.7]**	**100.0%**

Heterogeneity: $I^2 = 43\%$, $p = 0.12$

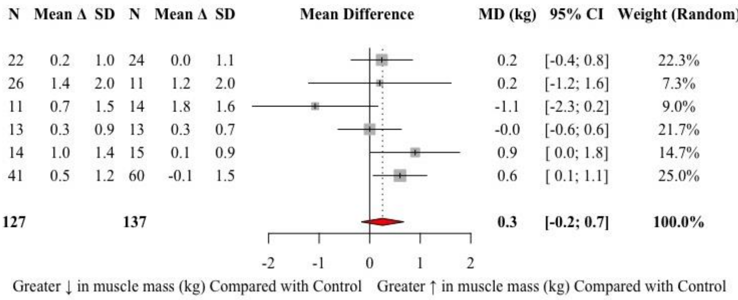

The mean difference between the change scores for high-intensity interval training (HIIT) and the control groups in kilograms
N = number of participants
Mean Δ = change score between pre- and post-tests
SD = standard deviation of the change score
MD = mean difference between the HIIT and control groups
CI = confidence interval
I^2 = variation across studies due to heterogeneity rather than chance

5. Forest plot of high-intensity interval training versus control for lean mass

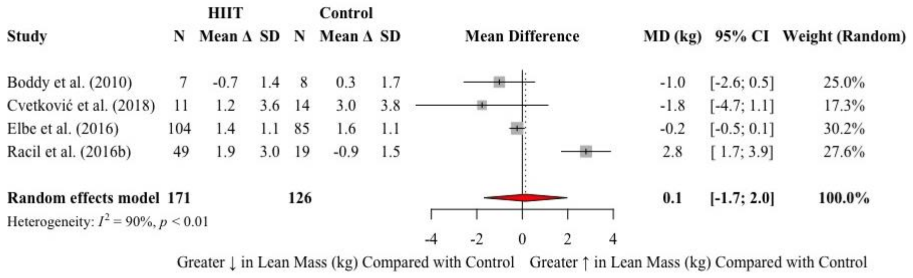

The mean difference between the change scores for high-intensity interval training (HIIT) and the control groups in kilograms
N = number of participants
Mean Δ = change score between pre- and post-tests
SD = standard deviation of the change score
MD = mean difference between the HIIT and control groups
CI = confidence interval
I^2 = variation across studies due to heterogeneity rather than chance

Cardiovascular Health Forest Plots

1. Forest plot of high-intensity interval training versus control for systolic blood pressure

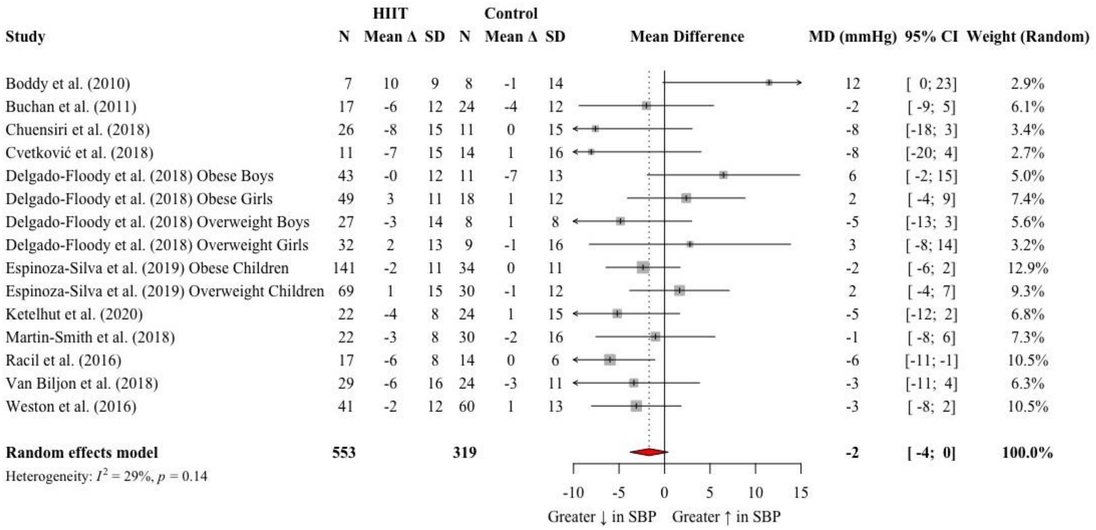

The mean difference between the change scores for high-intensity interval training (HIIT) and the control groups in millimetres of mercury.
N = number of participants
Mean Δ = change score between pre- and post-tests
SD = standard deviation of the change score
MD = mean difference between the HIIT and control groups
CI = confidence interval
SBP = systolic blood pressure
mmHg = millimetres of mercury
I^2 = variation across studies due to heterogeneity rather than chance

2. Forest plot of high-intensity interval training versus control for diastolic blood pressure

Study	HIIT N	HIIT Mean Δ	HIIT SD	Control N	Control Mean Δ	Control SD	MD (mmHg)	95% CI	Weight (Random)
Boddy et al. (2010)	7	6	5	8	-4	7	10	[4; 16]	6.0%
Buchan et al. (2011)	17	-2	7	24	-4	8	2	[-3; 7]	7.6%
Chuensiri et al. (2018)	26	-3	8	11	1	7	-4	[-9; 1]	7.0%
Cvetković et al. (2018)	11	-7	15	14	2	10	-9	[-19; 1]	3.6%
Delgado-Floody et al. (2018) Obese Boys	43	-2	9	11	-7	12	5	[-2; 12]	5.2%
Delgado-Floody et al. (2018) Obese Girls	49	3	10	18	-1	10	4	[-2; 9]	7.1%
Delgado-Floody et al. (2018) Overweight Boys	27	2	16	8	-3	18	5	[-9; 19]	2.2%
Delgado-Floody et al. (2018) Overweight Girls	32	0	10	9	1	11	-0	[-8; 7]	4.8%
Espinoza-Silva et al. (2019) Obese Children	141	1	9	34	-2	10	4	[0; 8]	8.6%
Espinoza-Silva et al. (2019) Overweight Children	69	1	14	30	-1	12	3	[-3; 8]	6.9%
Ketelhut et al. (2020)	22	-2	7	24	0	9	-3	[-7; 2]	7.9%
Martin-Smith et al. (2018)	22	-1	13	30	2	10	-3	[-10; 4]	5.8%
Racil et al. (2016)	17	-6	4	14	-1	3	-5	[-7; -3]	10.0%
Van Biljon et al. (2018)	29	-3	8	24	0	7	-3	[-7; 1]	8.3%
Weston et al. (2016)	41	-6	9	60	-5	9	-1	[-5; 2]	8.9%
**Random effects model**	**553**			**319**			**0**	**[-2; 2]**	**100.0%**

Heterogeneity: $I^2 = 68\%$, $p < 0.01$

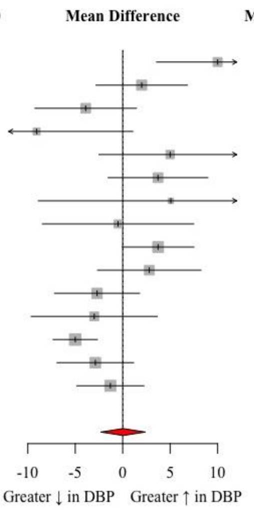

The mean difference between the change scores for high-intensity interval training (HIIT) and the control groups in millimetres of mercury.
N = number of participants
Mean Δ = change score between pre- and post-tests
SD = standard deviation of the change score
MD = mean difference between the HIIT and control groups
CI = confidence interval
DBP = diastolic blood pressure
mmHg = millimetres of mercury
I^2 = variation across studies due to heterogeneity rather than chance

3. Forest plot of high-intensity interval training versus control for resting heart rate

Study	HIIT N	HIIT Mean Δ	HIIT SD	Control N	Control Mean Δ	Control SD	MD (bpm)	95% CI	Weight (Random)
Chuensiri et al. (2018)	26	-8	10	11	0	11	-8	[-16; 0]	8.3%
Cvetković et al. (2018)	11	-11	12	14	5	8	-16	[-24; -8]	7.4%
Delgado-Floody et al. (2018) Obese Boys	43	-3	10	11	-3	12	-1	[-9; 7]	7.9%
Delgado-Floody et al. (2018) Obese Girls	49	1	12	18	-2	11	2	[-4; 9]	11.0%
Delgado-Floody et al. (2018) Overweight Boys	27	1	13	8	10	15	-9	[-21; 3]	4.4%
Delgado-Floody et al. (2018) Overweight Girls	32	-1	9	9	4	9	-5	[-11; 2]	10.4%
Gamelin et al. (2009)	22	-2	7	16	2	7	-3	[-8; 1]	14.8%
Racil et al. (2016a)	17	-3	2	14	0	2	-3	[-4; -2]	23.8%
Van Biljon et al. (2018)	29	-13	11	24	-6	10	-7	[-12; -1]	12.0%
**Random effects model**	**256**			**125**			**-5**	**[-7; -2]**	**100.0%**

Heterogeneity: $I^2 = 52\%$, $p = 0.03$

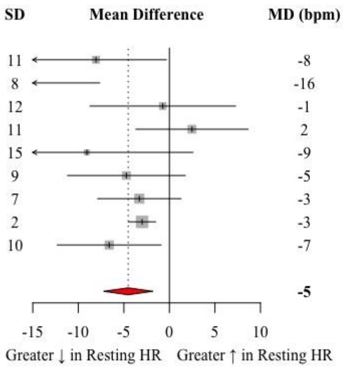

The mean difference between the change scores for high-intensity interval training (HIIT) and the control groups in beats per minute.
N = number of participants
Mean Δ = change score between pre- and post-tests
SD = standard deviation of the change score
MD = mean difference between the HIIT and control groups
CI = confidence interval
HR = systolic blood pressure
Bpm = beats per minute
I^2 = variation across studies due to heterogeneity rather than chance

Blood Profile Forest Plots

1. **Forest plot of high-intensity interval training versus control for fasting glucose**

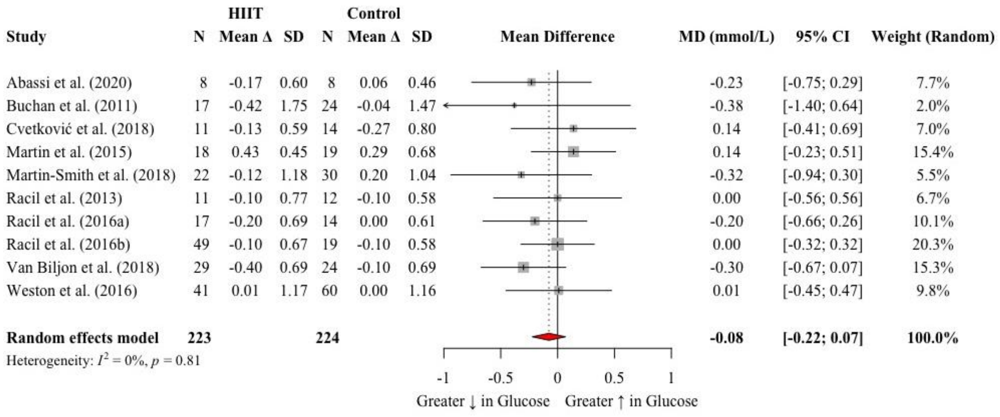

The mean difference between the change scores for high-intensity interval training (HIIT) and the control groups in millimoles per litre.
N = number of participants
Mean Δ = change score between pre- and post-tests
SD = standard deviation of the change score
MD = mean difference between the HIIT and control groups
CI = confidence interval
Mmol/L = millimoles per litre
I^2 = variation across studies due to heterogeneity rather than chance

2. **Forest plot of high-intensity interval training versus control for fasting insulin**

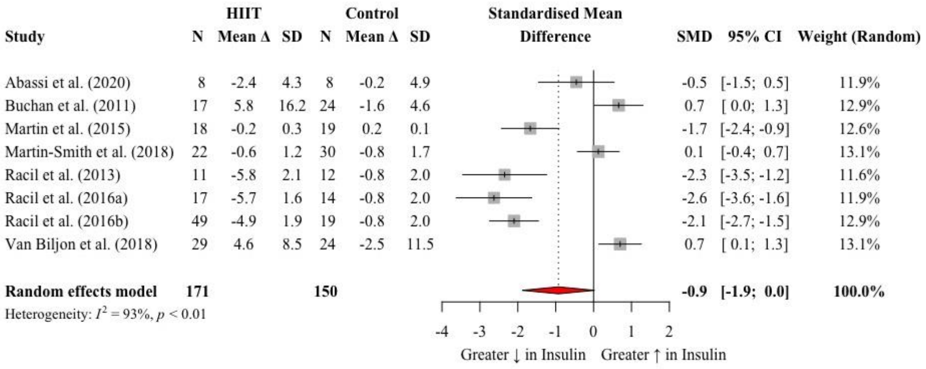

The standardised mean difference (SMD) between the change scores for high-intensity interval training (HIIT) and the control groups. SMD was used to account for different units of measurement presented.
N = number of participants
Mean Δ = change score between pre- and post-tests
SD = standard deviation of the change score
SMD = standardised mean difference between the HIIT and control groups
CI = confidence interval
I^2 = variation across studies due to heterogeneity rather than chance

3. **Forest plot of high-intensity interval training versus control for homeostatic model assessment – insulin resistance (HOMA-IR)**

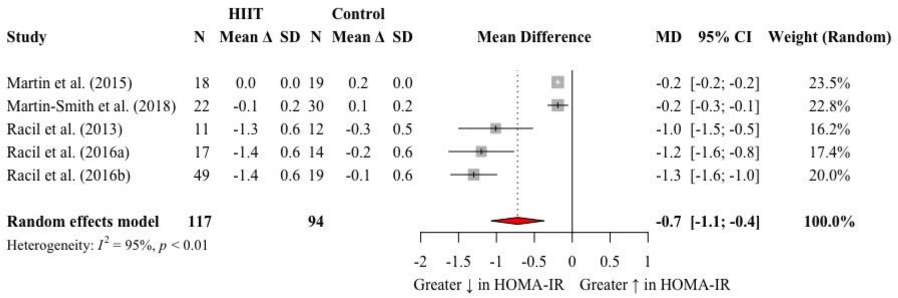

The mean difference between the change scores for high-intensity interval training (HIIT) and the control groups.
N = number of participants
Mean Δ = change score between pre- and post-tests
SD = standard deviation of the change score
MD = mean difference between the HIIT and control groups
CI = confidence interval
HOMA-IR = homeostatic model assessment – insulin resistance
I^2 = variation across studies due to heterogeneity rather than chance

4. **Forest plot of high-intensity interval training versus control for triglycerides**

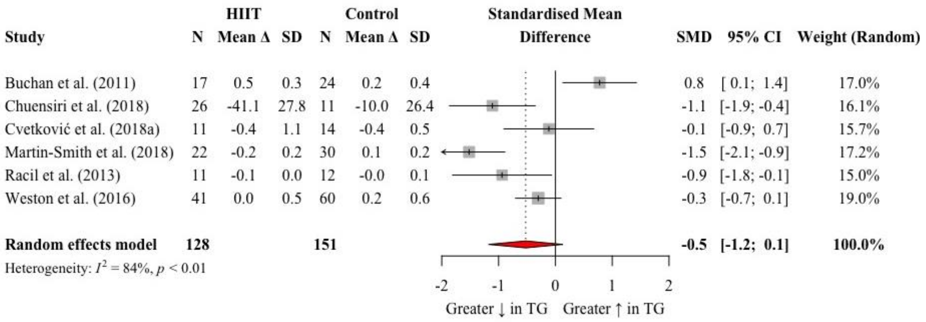

The standardised mean difference (SMD) between the change scores for high-intensity interval training (HIIT) and the control groups. SMD was used to account for different units of measurement presented.
N = number of participants
Mean Δ = change score between pre- and post-tests
SD = standard deviation of the change score
SMD = standardised mean difference between the HIIT and control groups
TG = triglycerides
CI = confidence interval
I^2 = variation across studies due to heterogeneity rather than chance

5. **Forest plot of high-intensity interval training versus control for total cholesterol**

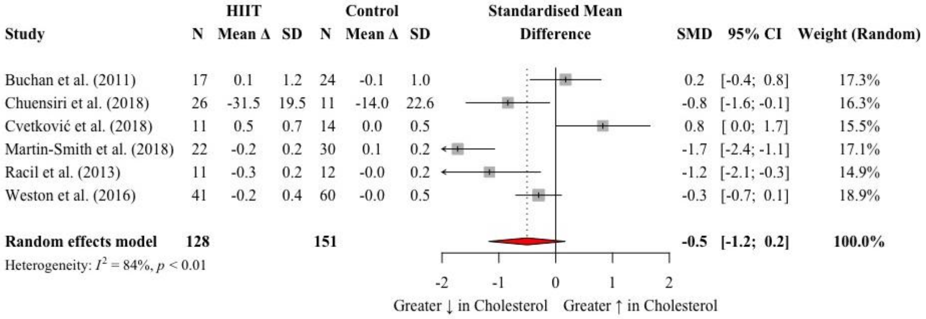

The standardised mean difference (SMD) between the change scores for high-intensity interval training (HIIT) and the control groups. SMD was used to account for different units of measurement presented.
N = number of participants
Mean Δ = change score between pre- and post-tests
SD = standard deviation of the change score

SMD = standardised mean difference between the HIIT and control groups
CI = confidence interval
I^2 = variation across studies due to heterogeneity rather than chance

6. Forest plot of high-intensity interval training versus control for total high-density lipoprotein

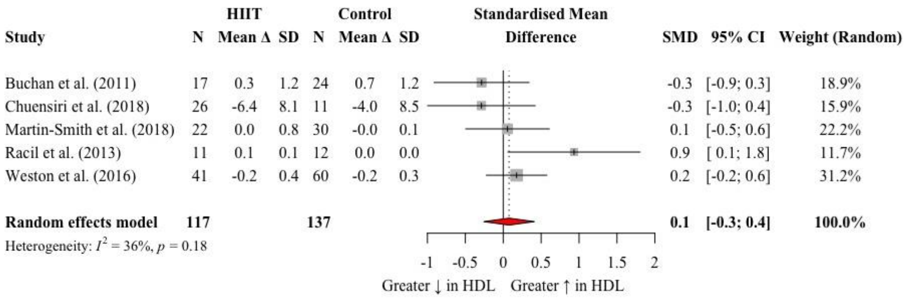

The standardised mean difference (SMD) between the change scores for high-intensity interval training (HIIT) and the control groups. SMD was used to account for different units of measurement presented.
N = number of participants
Mean Δ = change score between pre- and post-tests
SD = standard deviation of the change score
SMD = standardised mean difference between the HIIT and control groups
HDL = high-density lipoprotein
CI = confidence interval
I^2 = variation across studies due to heterogeneity rather than chance

7. Forest plot of high-intensity interval training versus control for total low-density lipoprotein

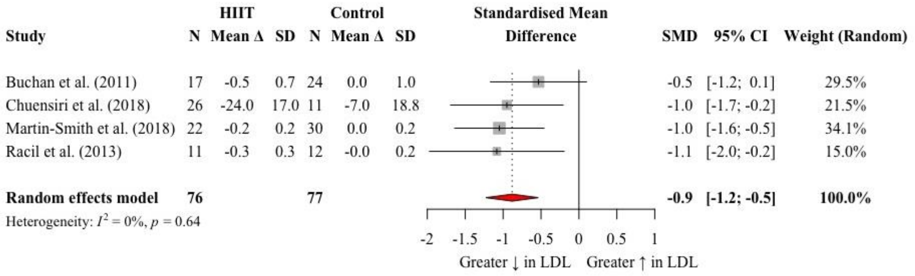

The standardised mean difference (SMD) between the change scores for high-intensity interval training (HIIT) and the control groups. SMD was used to account for different units of measurement presented.
N = number of participants
Mean Δ = change score between pre- and post-tests
SD = standard deviation of the change score
SMD = standardised mean difference between the HIIT and control groups
LDL = low-density lipoprotein
CI = confidence interval
I^2 = variation across studies due to heterogeneity rather than chance

Aerobic and Muscular Fitness Forest Plots

1. **Forest plot of high-intensity interval training versus control for all measurements of cardiorespiratory fitness**

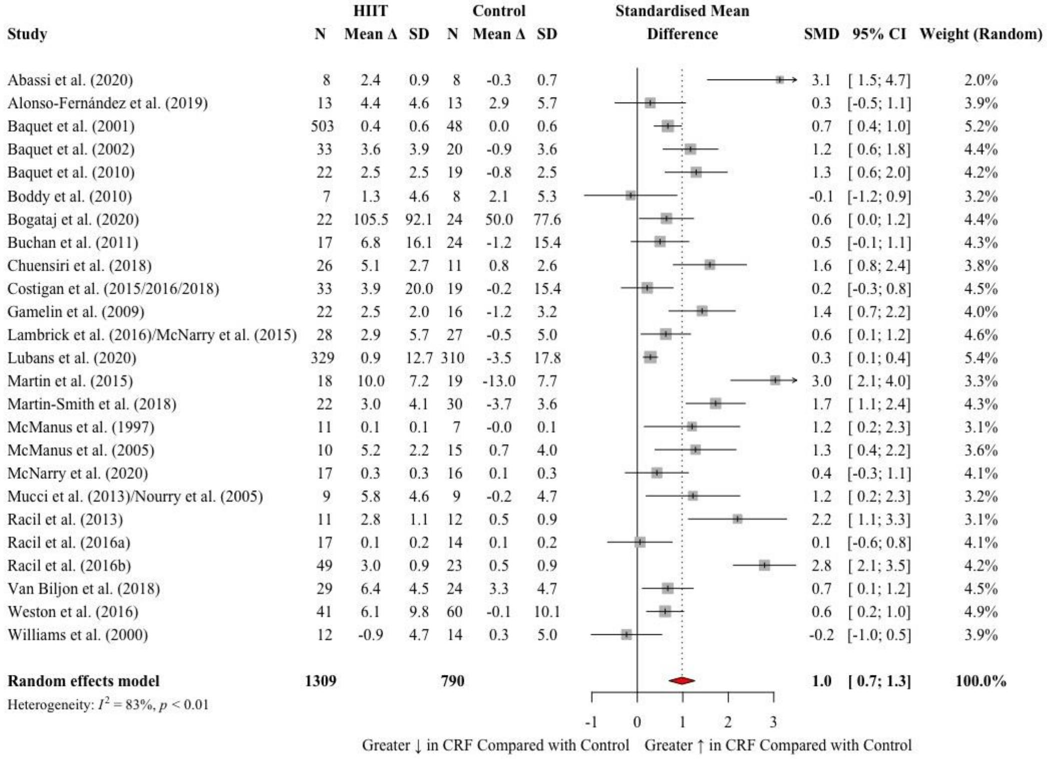

The standardised mean difference (SMD) between the change scores for high-intensity interval training (HIIT) and the control groups. SMD was used to account for different measures used to quantity cardiorespiratory fitness.
N = number of participants
Mean Δ = change score between pre- and post-tests
SD = standard deviation of the change score
SMD = standardised mean difference between the HIIT and control groups
CI = confidence interval
CRF = cardiorespiratory fitness
I^2 = variation across studies due to heterogeneity rather than chance

2. **Forest plot of high-intensity interval training versus control for cardiorespiratory fitness measured using a metabolic cart to measure relative VO_2**

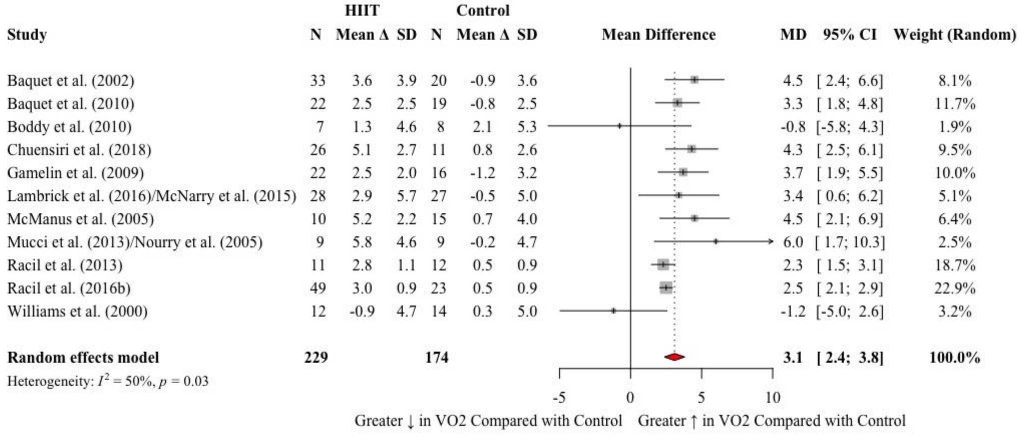

The mean difference between the change scores for high-intensity interval training (HIIT) and the control groups in ml/min/kg.
N = number of participants
Mean Δ = change score between pre- and post-tests

SD = standard deviation of the change score
MD = mean difference between the HIIT and control groups
VO_2 = maximum rate of oxygen consumption
I^2 = variation across studies due to heterogeneity rather than chance

3. Forest plot of high-intensity interval training versus control for cardiorespiratory fitness measured using number of shuttles completed in the 20 m shuttle run test

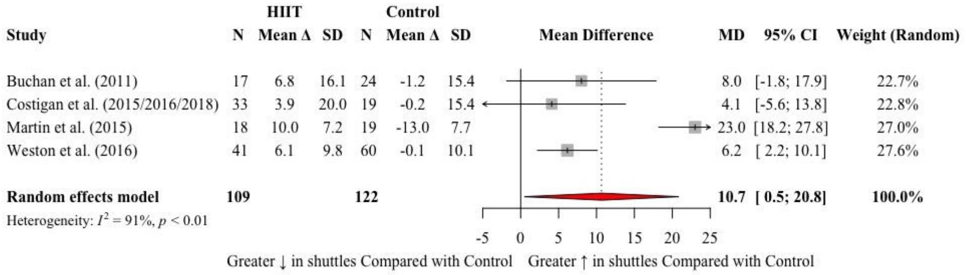

The mean difference between the change scores for high-intensity interval training (HIIT) and the control groups in number of shuttles.
N = number of participants
Mean Δ = change score between pre- and post-tests
SD = standard deviation of the change score
MD = mean difference between the HIIT and control groups
I^2 = variation across studies due to heterogeneity rather than chance

4. Forest plot of high-intensity interval training versus control for standing long jump

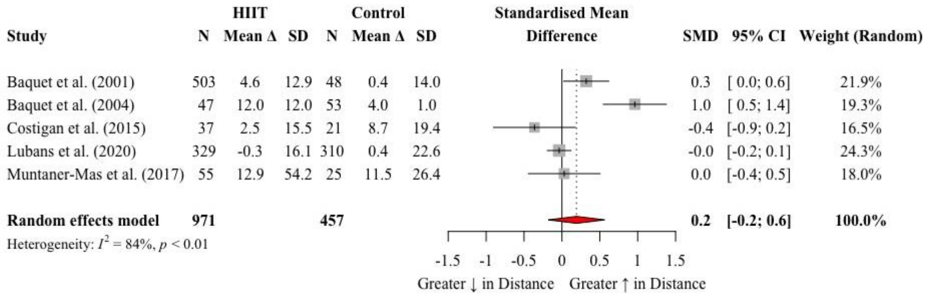

The standardised mean difference (SMD) between the change scores for high-intensity interval training (HIIT) and the control groups.
Mean Δ = change score between pre- and post-tests
SD = standard deviation of the change score
SMD = standardised mean difference between the HIIT and control groups
CI = confidence interval
I^2 = variation across studies due to heterogeneity rather than chance

5. Forest plot of high-intensity interval training versus control for countermovement jump

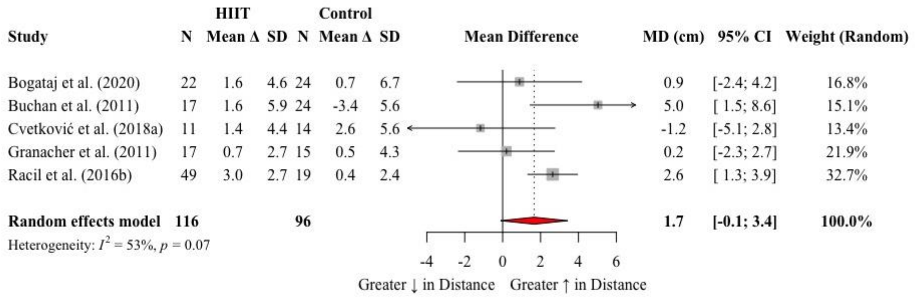

The mean difference between the change scores for high-intensity interval training (HIIT) and the control groups in centimetres
N = number of participants
Mean Δ = change score between pre- and post-tests

SD = standard deviation of the change score
MD = mean difference between the HIIT and control groups
CI = confidence interval
I^2 = variation across studies due to heterogeneity rather than chance
