# Supplementary material for: School-based high-intensity interval training programs in children and adolescents: A systematic review and meta-analysis
Source: PLoS One. 2022 May 4;17(5):e0266427. doi: 10.1371/journal.pone.0266427 (PMC9067698; doi:10.1371/journal.pone.0266427)
Supplement: Attachment — Submitted filename: ResponsetoReviewers.docx [file pone.0266427.s006.docx]

25 February 2022

Dear editor and reviewers,

**RE: REVISION PONE-D-22-01744 "School-based high-intensity interval training programs in children and adolescents: A Systematic Review and Meta-Analysis"**

Thank you for your thorough review of our article and the opportunity to address your comments. In this point-by-point response, we repeat your comments and present our responses in **bold**. We highlight edits to the manuscript text that were made in response to your comments in yellow, which we repeat in this document, where appropriate.

We look forward to hearing your thoughts on our revised manuscript.

Kind regards,

The authors

**Reviewer #1**

Comments to the Author:

1. This study performed a systematic review and meta-analysis on the physiological effects of high intensity interventions on school aged children. This is an important and needed study. The authors should be commended for using PRISMA and a priori registering this protocol. Overall, this is a well- designed, performed, and written study. Please see minor points below.

**Thank you for acknowledging the relevance of our paper and for your useful suggestions. We hope you find our responses below meet your expectations.**

Introduction:

1. Overall, the introduction reads well. I commend the authors for including previous systematic reviews and meta-analyses and why this specific SR and meta needs to be performed. This provides greater transparency and an improved foundational argument for this specific study.

**Thank you very much for your comments on the introduction and for acknowledging the usefulness of this systematic review and meta-analysis.**

Methods:

1. I commend the authors for using PRISMA and a priori registering this study. I also commend the authors for including the full search strategy as an appendix for reproducibility, and the use of a medical librarian.

**Thank you for acknowledging the methodological rigour of our manuscript.**

2. Can you clarify why quasi-experimental studies were included?

**Thank you for this question. We chose to include quasi-experimental studies in this review as we are aware that it is not always feasible to randomise schools, classes, or students in school-based intervention studies and we wanted to ensure that we included the full scope of research that currently exists in this setting. We have included clarification in our manuscript as follows on page 6, lines 65 – 67:**

*Both randomised control trials (RCTs) and quasi-experimental studies were included as randomisation is not always feasible in school-based studies and informative literature could have been missed if only RCTs were included.*

3. Suggest excluding fixed effects reporting as this may confuse the reader.

**We thank you for the suggestion. We have removed the fixed effects from the forest plots in the supporting file and have removed this statement in our methods section as follows on page 8, lines 118 and 119:**

*Random effect models were used to allow for variations between studies., ~~but we reported both fixed and random summary effect sizes in the figures~~.*

4. I commend the authors for the overall well designed meta-analyses and meta-regression.

**Once again, we thank you for acknowledging the methodological rigour of this review.**

Results

1. The risk of bias table is a bit busy and hard to read. Suggest splitting the table into A.B.C. tables for different RoB tools.

**Thank you for this recommendation. We have split Table 3 into two parts with one focused on randomised control trials and the other on the quasi-experimental studies. Please refer to Table 3 for these changes.**

2. Suggest adding at least one forest plot to the main results to improve readability and interpretation for the lay reader.

**We had previously considered including a forest plot in the main results. However, we were unable to give preference to one forest plot over the others and therefore, chose to include them all equally in a supporting file that could be viewed at the same time as the results by readers. We believe all the key information from the forest plots (main effect, heterogeneity, number of studies) is provided in Table 4 and we have provided an in-depth figure caption under each forest plot in the supporting file for readers to aid in interpretation.**

Discussion

1. Overall, the main summary is well written.

**Thank you very much for this comment.**

**Reviewer #2**

Comments to the Author:

The authors present a very interesting review of the works concerning HIIT applied in school age. I absolutely agree with the conclusions as this scheme could be more effective and pleasing to the kids.

**Thank you for taking time to provide feedback and for the acknowledging the usefulness of this review. We hope that it informs future school-based HIIT research and further integration of this research into the school context.**

In my opinion some aspects should be underlined:

1. Divide the studies into two age groups 5-12 and 12-17 as due to puberty the response to exercise and body composition are different

**Thank you for this suggestion. We agree that age may affect some of the responses; however, we are hesitant to categorise age into only 2 groups as changes related to age are ongoing, puberty occurs at different time points for different individuals, and there are clear sex differences for puberty. We have included age in our meta-regressions, where we had the statistical power to do this, using the mean age of the study participants as a continuous variable. Age was not statistically significant in any of our meta-regressions. This is most likely due the amount of variation between studies. We have added the following line to our discussion on page 35, lines 424 – 427:**

*As the body of evidence grows, it will be important to investigate potential sex and pubertal differences.* *Future studies should ensure that they report participants’ pubertal stages in addition to their sex.*

2. Also consider the difference in sex

**We once again thank you for this suggestion. Similar to our analysis with age, we included sex in our meta-regressions, where we had the power to investigate its effects. We did note differences based on sex as mentioned in section 3.4 of our results, where studies with a higher percentage of girls were associated with a greater decrease in body fat percentage and BMI, but not other variables. Unfortunately, most studies also only report the main effect and do not stratify their samples by sex. Therefore, we have added the following to our discussion on page 35, lines 424 – 428:**

*As the body of evidence grows, it will be important to investigate potential sex differences and pubertal differences. Future studies should ensure that they report participants’ pubertal stages in addition to their sex.* *Further, it will be important for future studies to report results stratified by sex and maturity status to enable the effects of these variables to be understood.*

3. It would be interesting to have a table with a summary of the most frequently used schemes, perhaps suggesting some that can be used, both in practice and in future studies.

**Thank you for this suggestion. We agree that it would be useful to know the most frequently used HIIT workouts and which could be useful in the future. However, there were considerable differences between most HIIT protocols as it is possible to alter mode, interval lengths, and sets. While this makes it difficult to propose suggested HIIT workouts for future use, it is also considered a benefit of HIIT workouts as they can enable endless variation to engage and appropriately challenge participants. We previously summarised the intervention length and frequency in Table 1, but we have added additional comments regarding the most frequent intervals and mode of exercise in the current literature to the results on page 10, lines 153 and 154:**

*The most common modality was running, and interval lengths within the interventions spanned from 10 seconds to a 4-minute bout of HIIT games.*

**Further, we added the following line into the section on process outcomes in the discussion on page 34, lines 390 to 395:**

*It will be important for future studies to appropriately document the attendance and fidelity of these interventions for proper evaluation. This could help inform readers of HIIT protocols that are more likely to achieve high intensity in this setting. The intervention timing and facilitators varied between studies, and this could have implications on the reach, maintenance, and scalability of studies. However, the variation in the HIIT protocols across studies suggests that there are opportunities to tailor protocols to specific classes or students to appropriately engage and challenge them, and in turn optimise associated outcomes.*

4. A correlation with the countries where the studies were carried out would also be interesting since, unfortunately, the practice and attention to physical activity is not uniform.

**This would be very interesting to investigate. However, 60% of the studies were conducted in Europe and we don’t believe there is the statistical power to properly investigate this at this point in the literature. Further, there is a large amount of variability within countries, school systems, and individual schools. However, we have attempted to highlight the need for more contextual information around this using the following sentence in our discussion on page 35, line 428 – 431:**

*Additionally, beyond sex and maturity, studies should aim to investigate if these health outcomes are present across schools in different contexts with varying physical activity policies and practices as these vary greatly between countries, school systems, and individual schools.*

5. Finally, a stratification based on the assessment of body composition would be interesting, which may go beyond the BMI (for example plicometry or bioimpedance)

**Thank you for this suggestion. For body fat percentage, where we had the power to examine different assessments, we included the assessment in categories (DXA, bioimpedance, estimate from skinfold, or not reported) in a meta-regression and we report the following at the end of the section on physical health outcomes (page 23, line 240 and page 25, lines 252 and 253):**

*The method used to assess CRF (20 m shuttle run, cycle ergometer, or treadmill ergometer) and body fat percentage (Dual X-ray absorptiometry, bioelectrical impedance, or skinfold estimation) did not significantly moderate the results.*

**Reviewer #3**

Comments to the Author:

1. You have provided a comprehensive review of the included papers, and I commend your analysis techniques.

**Thank you very much for taking time to provide feedback and for acknowledging the comprehensiveness of the review.**

2. As you have declared that a large majority of the selected papers show a high risk of bias (line 202) this should be reflected in your conclusion (line 448 onwards), and you may feel it appropriate to make recommendations for future studies (line 453 onwards) to overcome these issues of bias.

**Thank you for this useful suggestion. We have modified our conclusion on page 37, line 468, as follows:**

*HIIT is an effective strategy for improving various health outcomes within the school setting, with our meta-analyses indicating meaningful improvements in markers of body size and composition, cardiovascular disease blood biomarkers, and CRF when compared to a non-exercise control group. However, our risk of bias results highlight that more high-quality studies are needed in this area.*

**We have also added a sentence to the future direction section (page 35, lines 404 - 407) about future studies:**

*Future studies should focus on 1) providing justification for their sample size; 2) reporting adherence, fidelity, and whether blinding occurred to determine deviations from the intended intervention; 3) and performing statistical analyses that account for any missing data.*

3. Please also explain the exceptional situation that restricts data availability.

**We have modified our data availability statement. We have now included our dataset in our submission through a supporting information file.**
